# A prospective randomised, controlled clinical trial comparing medial and lateral entry pinning with lateral entry pinning for percutaneous fixation of displaced extension type supracondylar fractures of the humerus in children

**DOI:** 10.1186/1749-799X-7-6

**Published:** 2012-02-15

**Authors:** Abhijan Maity, Debasish Saha, Debasis Sinha Roy

**Affiliations:** 1 Department of Orthopaedics, Burdwan Medical College, Burdwan, West Bengal, India; 2 Department of Anaesthesiology, Burdwan Medical College, Burdwan, West Bengal, India; 3 c/o Mr. Kalipada Maity, Bhatchala (Near Banka Bridge), PO Sripally Dist., Burdwan, West Bengal, India PIN-713103

**Keywords:** Supracondylar fractures, Humerus, Children, Percutaneous fixation

## Abstract

**Objective:**

To compare the efficacy of medial and lateral entry pinning with lateral entry pinning for percutaneous fixation of displaced (Gartland type II and type III) extension type supracondylar fractures of the humerus in children.

**Methods:**

The study was a single center, prospective, randomized controlled clinical trial. Between October 2007 and September 2010, 160 patients who satisfy the inclusion and exclusion criterias were enrolled in the study, with 80 patients in each group. All the percutaneous pinning was done according to a uniform standardized technique. The patients were re-evaluated as outpatients at three weeks, six weeks and three months after the surgery. At three months follow-up visit, following informations were recorded as outcome measures: (i) Carrying angle (deg) (ii) passive range of elbow motion (deg) (iii) Flynn's criteria for grading, based on the loss of carrying angle and loss of total range of elbow motion. (iv) Baumann angle (deg) (v) Change in Baumann angle (deg) between the Intraoperative radiographs after the surgery and radiographs at three months follow-up visit (vi) loss of reduction grading, based on the change in the Baumann angle.

**Results:**

There were no significant differences between the two groups with regard to base-line characteristics, withdrawals and complication rate. At three months follow-up visit, patients were evaluated by recording the various outcome measures. There were no significant differences between the two groups with regard to the various outcome measures such as carrying angle, passive range of elbow motion, Flynn grading, Baumann angle, change in the Baumann angle and loss of reduction grading.

**Conclusions:**

If a uniform standardized operative technique is followed in each method, then the result of both the percutaneous fixation methods will be same in terms of safety and efficacy.

## Introduction

Supracondylar fractures of the humerus are the most common fractures about the elbow in children [1,2]. According to Boyd and Altenberg [3], these fractures account for 65.4% of upper extremity fractures in children. Gartland [4] proposed a classification for these fractures: type I, Undisplaced; type II, displaced with the posterior cortex intact; and type III, completely displaced with no cortical contact. Supracondylar fractures may be associated with a number of complications such as neurovascular injuries, malunion, compartment syndrome, iatrogenic neurovascular injury and elbow stiffness [1,2,5]. Cubitus varus due to malunion is the most common angular deformity and the incidence varies from 5% according to Flynn et al. [6], to 21% according to Arino et al. [7]. Incidence of iatrogenic ulnar nerve injuries after percutaneous fixation with medial and lateral entry pinning was about 15% according to Chai [8].

The recommended method of treatment for displaced (Gartland [4] type II and type III) extension type Supracondylar fractures of the humerus in children is closed reduction and percutaneous pin fixation. But, the optimal method of percutaneous pin fixation varies among authors. Swenson [9], Casiano [10] and Flynn et al. [6] used two crossed pins, one introduced medially and one laterally. Arino et al. [7] used two lateral pins.

Though crossed medial-lateral pin fixation provides increased biomechanical stability, but simultaneously it carries the risk of iatrogenic ulnar nerve injury from placement of the medial pin [11-13]. Conversely, the two- lateral pin fixation avoids the danger of iatrogenic ulnar nerve injury, but it provides less biomechanical stability [14-18].

The aim of our present study was to compare the efficacy of medial and lateral entry pinning with lateral entry pinning for percutaneous fixation of displaced (Gartland [4] type II and type III) extension type supracondylar fractures of the humerus in children.

## Materials and methods

### Trial designs

The study was a single center, prospective, randomized controlled clinical trial, conducted in Department of Orthopaedics and Traumatology of our institution from October 2007 to September 2010. The protocol was approved by the ethics committee of our institution. Randomization was done after we had taken written informed consent from the study participants and obtained base line information. The random assignment scheme was created from a table of random numbers. Opaque prenumbered sealed envelopes containing random assignments were maintained by the hospital pharmacist.

### Patients

All the children attending in the Accident and Emergency Department or, in the Outpatient Department of Orthopaedics and Traumatology in our institution between October 2007 and September 2010 with supracondylar fractures of the humerus were enrolled in the present study if they had the following inclusion criterias: (i) age between two and twelve years (ii) Unilateral fracture (iii) Extension type (iv) Displaced Gartland [4] type II and type III (v) presenting within seventy two hours after the injury (vi) no other associated injury in the same limb (vii) no previous fracture in the same limb.

Patients were excluded if they fulfill the following exclusion criterias: (i) age less than two years or, greater than twelve years (ii) Bilateral fracture (iii) Flexion type (iv) Undisplaced Gartland [4] type I (v) presenting more than seventy two hours after the injury (vi) associated injury in the same limb (vii) Previous fracture in the same limb (viii) open fracture (ix) unsatisfactory closed reduction requiring open reduction (x) floating elbow (xi) failure to perform a preoperative neurovascular examination (xii) associated neurovascular injury requiring surgical exploration

### Treatment

Surgery was done under general anesthesia. All the patients were positioned supine on a fracture table and closed reduction were performed under the fluoroscopic guidance. If the closed reduction was satisfactory, then percutaneous fixation with either crossed medial-lateral pin or, two-lateral pin was done according to the random assignment scheme.

All the crossed medial-lateral pinning was done according to the mini-open technique described by Green et al. [19]. All the lateral entry pinning was done according to the technique described by Aroson and Prager [20].

**Figure 1:**
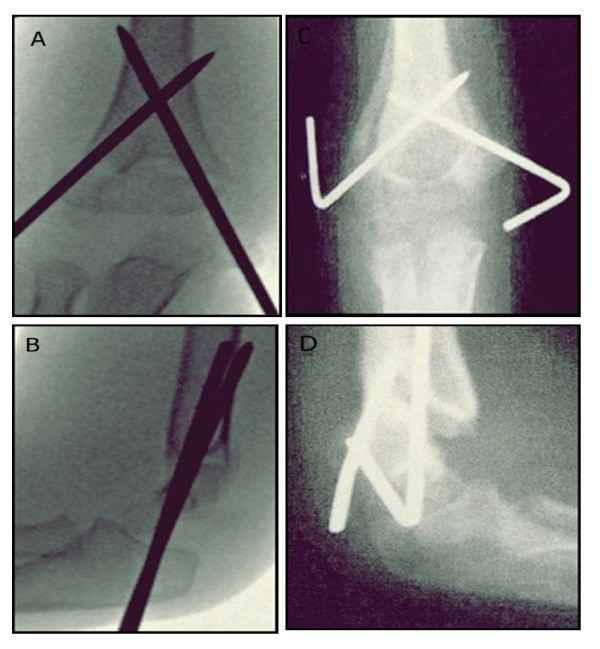
**A, Immediate postoperative Anteroposterior radiograph of crossed medial-lateral entry pinning showing acceptable reduction**. **B**, Immediate postoperative lateral radiograph of crossed medial-lateral entry pinning showing acceptable reduction. **C**, Follow-up Anteroposterior radiograph showing loss of reduction in crossed medial-lateral entry pinning. **D**, Follow-up lateral radiograph showing loss of reduction in crossed medial-lateral entry pinning.

**Figure 2:**
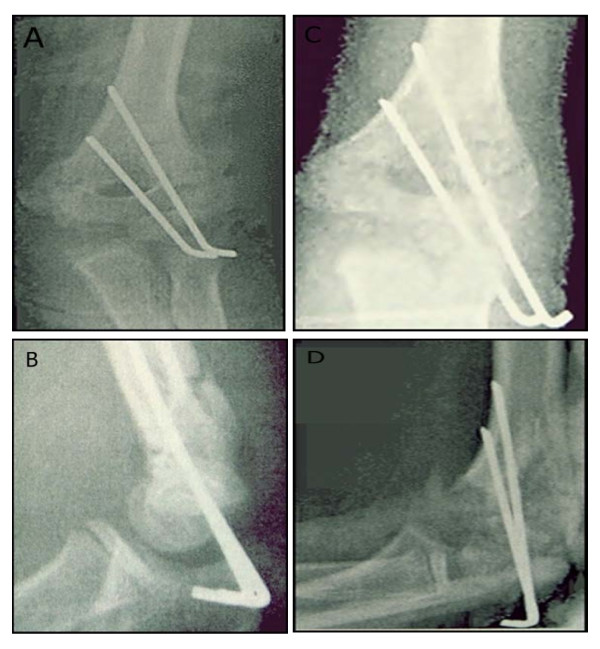
**A, Immediate postoperative Anteroposterior radiograph of two-lateral entry pinning showing acceptable reduction**. **B**, Immediate postoperative lateral radiograph of two-lateral entry pinning showing acceptable reduction. **C**, Follow-up Anteroposterior radiograph showing loss of reduction in two-lateral entry pinning. **D**, Follow-up lateral radiographs showing loss of reduction in two-lateral entry pinning.

Intraoperative radiographs obtained after pin placement was accepted in all cases. Loss of reduction were observed in postoperative radiographs of nine children who underwent crossed medial-lateral pin fixation as shown in Figure 1 and in postoperative radiographs of eight children who underwent two lateral pin fixation as shown in Figure 2.

Pin-tract infection was defined as documentation of purulent or, seropurulent discharge/erythema around the pin-site, with or without bacteriological evidence of infection.

Betadine soaked gauzes were placed around each pin site and a kling flexible dressing bandage was then used as an occlusive dressing around all the pin sites. The dressing was left in place for 48 hours. Thereafter, each pin site was cleaned with normal saline for removal of crust. In the presence of exudates, a light gauze dressing was applied. In the absence of exudates, the pin sites were left uncovered. A single preoperative parenteral dose of Cefuroxime was given at the time of induction, which was continued post-operatively for 72 hours. The patient was discharged with advice of taking oral antibiotics only if a pin site infection was developed. The signs and symptoms of a pin site infection were clearly explained to the parents (pain, erythema, tenderness, discharge). Parents were instructed to clean the pin sites daily with cotton swabs soaked in normal saline to remove all crusts.

### Follow-up and outcome measure

The patients were re-evaluated as outpatients at three weeks, six weeks and three months after the surgery. Follow-up assessment of each patient was done by the same doctor throughout the trial. Both the surgeons and the patients were not blinded of the treatment received throughout the trial. Plaster cast and pins were removed at three weeks. At three months follow-up visit, following informations were recorded as outcome measures: (i) Carrying angle (deg) (ii) passive range of elbow motion (deg) (iii) Flynn's criteria for grading [6], based on the loss of carrying angle and loss of total range of elbow motion (Table 1). (iv) Baumann angle (deg), calculated on the skiagram of anteroposterior view of elbow with the method described by Williamson et al. [21]. (v) Change in Baumann angle (deg) between the Intraoperative radiographs after the surgery and radiographs at three months follow-up visit (vi) loss of reduction grading, based on the change in the Baumann angle according to the method described by Skaggs et al. [22] (Table 2).

**Table 1:** Flynn's criteria for grading [6]

Result	Rating	Carrying angle loss (Degrees)	Total range of elbow motion loss (Degrees)
Satisfactory	Excellent	0-5	0-5

	Good	5-10	5-10

	Fair	10-15	10-15

Unsatisfactory	Poor	Over 15	Over 15

**Table 2:** Skaggs et al. [22] criteria for grading loss of reduction

Change in Baumann angle	Loss of reduction grading
< 6°	None

6°-12°	Mild

> 12°	Major

### Source of funding

There was no source of external funding in support of this study.

### Sample size

We selected the major loss of reduction (defined as a change in the Baumann angle of > 12° between the Intraoperative radiographs and radiographs at three months follow-up visit) as the primary outcome measure at three months. We estimated that, in order to detect a 15% difference in the rates of major loss of reduction (with a two-sided alpha value of 5%, a statistical power of 80%) between the two groups, at least sixty patients had to be recruited in each group. We therefore planned to enroll 80 patients in each group considering for an expected maximum withdrawal rate of 25 percent.

### Statistical analysis

All statistical analysis was based on an "intention to treat" principle; therefore patients who withdrew from the study, the data at the time of withdrawal were carried forward to all subsequent evaluations. The outcomes of treatment with the crossed medial-lateral pin were compared with those of treatment with the two-lateral pin with the use of parametric and nonparametric analyses as appropriate for the data. The independent-sample student t tests, Fisher's exact test, Pearson chi-square tests, Mann-Whitney U test were performed with use of SAS statistical package (SAS institute, Cary, North Carolina). A p value of < 0.05 was considered to be statistically significant.

## Results

### Study group

Between October 2007 and September 2010, 160 patients who satisfy the inclusion and exclusion criterias were enrolled in the study, with 80 patients in the crossed medial-lateral pin entry group and 80 in the two lateral pin entry group. There were no significant differences between the two groups regarding base-line characteristics such as age, male sex, side, types of displacement, types of fracture, interval from injury to admission and interval from admission to surgery (Table 3).

**Table 3:** Base-line characteristics of 160 patients with displaced (Gartland [4] type II and III) extension type supracondylar fractures of humerus randomly assigned to receive percutaneous fixation with either crossed medial-lateral pin or, two-lateral pins

Baseline Characteristics	Crossed medial-lateral pin entry group (n = 80)	Two-lateral pin entry group (n = 80)	p value
**Age †(years)**	6.24 ± 1.77	6.12 ± 1.82	0.67ζ

**Male sex ¶(% of patients):**	48 (60)	51 (64)	0.74ψ

**Side¶(% of patients):**			0.53 ψ

**Left**	68(85)	64(80)	

**Right**	12(15)	16(20)	

**Types of Displacement¶ (% of patients):**			0.71Ø

**Posterolateral**	38(48)	33(41)	

**Posteromedial**	24 (30)	28 (35)	

**Posterior**	18 (22)	19 (24)	

**Types of fracture according to Gartland **[4]**¶ (% of patients):**			0.75 ψ

**Type II**	34(42)	37(46)	

**Type III**	46(58)	43(54)	

**Interval from admission to surgery† (hours)**	25.4 ± 10.26	23 ± 8.78	0.11ζ

**Interval from injury to admission† (hours)**	27.8 ± 16.12	29.47 ± 11.74	0.45ζ

† The datas are given as the mean ± standard deviation. ¶ The datas are given as the number (%) of patients. ζ Independent-sample student t test. ψ Fisher's exact. Ø Chi-square test

### Withdrawals

A total of 30 of the 160 patients did not complete the three follow-up visits. In the crossed medial-lateral pin entry group, 2 patients did not come after the surgery, 5 patients did not come after the first visit and 9 patients did not come after the second visit. In the two lateral pin entry group, 2 patients did not come after the first injection, 4 patients did not come after the first visit and 8 patients did not come after the second visit. So, only 130 of the 160 patients were available at three months follow-up visit. There were no significant differences between the two groups with regard to withdrawals (Table 4). Mean number of follow-up visits in the crossed medial-lateral pin entry group (2.7 ± 0.7) was not significantly different from that in the two lateral pin entry group (2.72 ± 0.67) (Independent sample student t test, p = 0.85).

**Table 4:** Withdrawals of the 160 patients with displaced (Gartland [4] type II and III) extension type supracondylar fractures of humerus randomly assigned to receive percutaneous fixation with either crossed medial-lateral pin or, two-lateral pins

Lost to follow-up	Crossed medial-lateral pin entry group (n = 80)	Two-lateral pin entry group (n = 80)	p value
**After the surgery ψ**	2 (3)	2 (3)	1.0ρ

**After the first visit ψ**	5 (6)	4 (5)	1.0ρ

**After the second visit ψ**	9 (11)	8 (10)	1.0ρ

ψ The datas are given as the number (%) of patients. ρ Fisher's exact test

### Complications

There were no significant differences between the two groups regarding neurovascular complications at the time of admission, iatrogenic ulnar nerve injury, and pin track infection (Table 5).

**Table 5:** Complications of the 160 patients with displaced (Gartland [4] type II and III) extension type supracondylar fractures of humerus randomly assigned to receive percutaneous fixation with either crossed medial-lateral pin or, two-lateral pins

Complications	Crossed medial-lateral pin entry group † (n = 80)	Two-lateral pin entry group* (n = 80)	p value
**Neurovascular complications at the time of admission ψ**			0.75¶

**Radial nerve injury**	6 (8)	5 (6)	

**Median nerve injury**	9 (11)	12 (15)	

**Pulseless pink hand**	7 (9)	6 (8)	

**Iatrogenic ulnar nerve injury ψ**	0	0	1.0ρ

**Pin track infection at three weeks follow-up visit ψ**	2 (3)	3 (4)	1.0ρ

ψ The datas are given as the number (%) of patients. ¶ Chi-square test. ρ Fisher's exact test

† Data were missing for 2 patients in case of pin track infection because they do not come after the surgery* Data were missing for 2 patients in case of pin track infection because they do not after the surgery

### Response to treatment

At three months follow-up visit, patients were evaluated by recording the various outcome measures. There were no significant differences between the two groups with regard to the various outcome measures such as carrying angle, passive range of elbow motion, Flynn [6] grading, Baumann angle, change in the Baumann angle and loss of reduction grading (Table 6).

**Table 6:** Comparative outcome measures at three months after the surgery in both groups

Outcome measure	Crossed medial-lateral pin entry group † (n = 64)	Two-lateral pin entry group *(n = 66)	p Value
**Carrying angle (degree)π**	5.52 ± 3.77	5.56 ± 4.62	0.95ζ

**Loss of Carrying angle (degree) π**	3.58 ± 3.08	3.86 ± 3.33	0.62ζ

**Passive range of elbow motion (degree) **π			

**Flexion**	128.3 ± 12.67	127.96 ± 4.38	0.75 ζ

**Extension**	-2.6 ± -0.13	-2.56 ± -0.16	0.12ζ

**Total range of motion**	130.58 ± 3.9	129.39 ± 4.48	0.11ζ

**Loss of total passive range of elbow motion (degree) **π	3.4 ± 2.9	3.8 ± 3.21	0.45 ζ

**Flynn grading (% of patients) **¶			0.84 ψ

**Excellent**	51(80)	48(73)	

**Good**	6(9)	8(12)	

**Fair**	7(11)	10(15)	

**Poor**	0	0	

**Loss of reduction grading (% of patients) **¶			0.94 ψ

**Major**	0	0	

**Mild**	9 (15)	8 (12)	

**None**	55 (85)	58 (88)	

**Baumann angle (degree)**π	77.2 ± 4.35	76.2 ± 3.51	0.15 ζ

**Change in the Baumann angle (degree) **π	3.57 ± 2.43	3.71 ± 2.1	0.72 ζ

† Data were missing for 16 patients * Data were missing for 14 patients

π The datas are given as the mean ± standard deviation

¶ The datas are given as the number (%) of patients

ζ Independent-sample student t test

ψ Chi-square test

## Discussion

The standard treatment for displaced (Gartland [4] type II and type III) extension type Supracondylar fractures of the humerus in children is closed reduction and percutaneous pin fixation. But, controversy persists among authors regarding optimal method of percutaneous pin fixation. Swenson [9], Casiano [10] and Flynn et al. [6] used two crossed medial-lateral pins. Arino et al. [7] used two lateral pins.

Though crossed medial-lateral pin configuration provides good biomechanical stability, but simultaneously it carries the increased risk of iatrogenic ulnar nerve injury due to placement of the medial pin [11-13]. Conversely, though the two- lateral pin configuration carries less risk of iatrogenic ulnar nerve injury, but it provides less biomechanical stability [14-18].

We performed a prospective randomized study to compare the efficacy of medial and lateral entry pinning with lateral entry pinning for percutaneous fixation of displaced (Gartland [4] type II and type III) extension type supracondylar fractures of the humerus in children.

In our study, we found no significant difference between the two groups with regard to iatrogenic ulnar nerve injury and loss of reduction grading.

Though seven studies [11,14,18,23-26] have been done so far to compare the efficacy of medial and lateral entry pinning with lateral entry pinning for percutaneous fixation of displaced (Gartland [4] type II and type III) extension type supracondylar fractures of the humerus in children but, it is very difficult to compare between them because: (i) pinning technique, pin size, position of elbow during pinning differs in various studies, (ii) only one study [11] consists of more than 50 patients in each group but, that was a retrospective study, (iii) Most of the studies are retrospective and uncontrolled [11,14,18,24,25]. Only two studies [23,26] are randomized controlled but, these studies consist of less than 50 patients in each group. All of these studies found no significant difference between the two methods in terms of loss of reduction and six studies found no significant difference between the two methods in terms of iatrogenic nerve injury. Only one shows significant difference in favor of lateral entry pinning method in terms of iatrogenic nerve injury. So, convincing evidence of the optimal method of percutaneous pin fixation is lacking in various literature overviews.

Brauer et al. [27] performed a systematic review using pooled data of 2054 children from 35 previous studies: 2 randomized trials, 6 retrospective studies and 25 case series. They found no significant difference between the two groups in terms of loss of reduction and iatrogenic nerve injury.

So, the results of our study are consistent with the results of most of the previous studies consists of the same clinically relevant question.

The major strength of the present study is its prospective randomized design. All of the patients in each group were operated on according to a uniform standardized well-accepted technique. Also, thorough follow-up assessment of each patient was done with the use of various clinical and radiological outcome measures at standardized intervals. Follow-up assessment of each patient was done by the same doctor throughout the trial.

The major limitation of our study is that, both the surgeon and the patients were not blinded of the treatment received throughout the trial. Another weakness of our study is the number of patients who did not complete the three-month follow-up visit. However, as the rate of the patients lost to follow-up in our study is comparable with that in other studies, we do not believe that it hampers our results.

In conclusion, we found that if a uniform standardized operative technique is followed in each method, then the result of both the percutaneous fixation methods will be same in terms of safety and efficacy.

## Competing interests

The authors declare that they have no competing interests.

## Authors' contributions

AM carried out the monitoring of data collection, patient follow-up, randomization etc. DS carried out the monitoring of operation under anaesthesia. DSR carried out the monitoring of surgical technique. All authors read and approved the final manuscript.
